# Bilateral retinal pigment epithelial tears in acute central serous chorioretinopathy without bullous retinal detachment: A case report

**DOI:** 10.1097/MD.0000000000027391

**Published:** 2021-10-01

**Authors:** Woo Hyuk Lee, Bum Jun Kim, Yong Seop Han

**Affiliations:** a Department of Ophthalmology, Gyeongsang National University Changwon Hospital, Changwon, Republic of Korea; b Department of Ophthalmology, Gyeongsang National University College of Medicine, Jinju, Republic of Korea; c Health Science Institute, Gyeongsang National University, Jinju, Republic of Korea.

**Keywords:** case report, central serous chorioretinopathy, retinal pigment epithelial tear

## Abstract

**Rationale:**

Several reports have described retinal pigment epithelial (RPE) tears in central serous chorioretinopathy (CSC). However, there have been no reports of spontaneously large RPE tears in acute CSC without bullous retinal detachment (RD). Herein, we report and provide sequential images of a case of bilateral spontaneous large RPE tears in patient with acute CSC without bullous RD.

**Patient Concerns:**

An 88-year-old female patient was admitted with impaired vision in both eyes, which began 10 days prior. The visual acuity was 0.4 and 0.5 in the right and left eye, respectively. She had started taking oral steroids 2 weeks prior for polymyalgia.

**Diagnosis:**

Ophthalmologic examinations, including fundus photography, optical coherence tomography, and fluorescence angiography, were performed, and she was diagnosed with steroid-induced acute CSC in both eyes with large pigment epithelial detachment (PED) of approximately 4-disc diameter. Discontinuation of steroids and follow-up ophthalmic examinations were performed. However, a spontaneous large RPE tear occurred in the right eye.

**Interventions:**

We performed follow-ups more frequently and CSC treatment such as laser photocoagulation, photodynamic therapy, and anti-vascular endothelial growth factor injections were not performed.

**Outcomes:**

Nine months later, a spontaneous large RPE tear occurred sequentially in the left eye. Her final visual acuity was 0.3 and 0.15 in the right and left eye, respectively.

**Lessons:**

Patients may spontaneously develop large RPE tears in both eyes, despite no treatment for acute CSC with non-bullous RD. Large PED and old age may affect this. Therefore, for a CSC patient with a large PED and advanced age, attention must be paid when determining treatment.

## 1 Introduction

Retinal pigment epithelial (RPE) tear is a pigment epithelial detachment (PED) complication that is known to cause a sudden loss of vision. Neovascular age-related macular degeneration (AMD) is the most common cause of RPE tears, and anti-vascular endothelial growth factor (VEGF) injections are also known to be related to RPE tear development.^[1,2]^ Moreover, its manifestations have also been reported with trauma, choroidal effusion, histoplasmosis, laser photocoagulation, retinal detachments, chorioretinal scars, metastatic carcinoma, Vogt-Koyanagi-Harada disease, and central serous chorioretinopathy (CSC).^[3–8]^ However, there have been no reports of spontaneous bilateral large RPE tears arising in acute CSC without bullous retinal detachment (RD). Herein, we report on and provide sequential images taken before and after bilateral large RPE tears in a case of acute CSC without bullous RD.

## 2 Ethics statement

The study was approved by the Ethics Committee of Gyeongsang National University Changwon Hospital, South Korea. The Patient has provided informed consent for publication of this case report and accompanying images.

## 3 Case report

An 88-year-old female patient was admitted with impaired vision in both eyes, which began ten days prior. Her visual acuity was 0.4 and 0.5 in the right and left eye, respectively. Her height and weight were 150 cm and 40 kg, respectively. She had a history of diabetes, hypertension, and polymyalgia, and she had started taking oral steroids 2 weeks prior for polymyalgia. Taken steroid dose was 15 mg/day, and this was the first time for using steroid. The patient underwent non-complicated cataract surgery in both the eyes.

**Figure 1:**
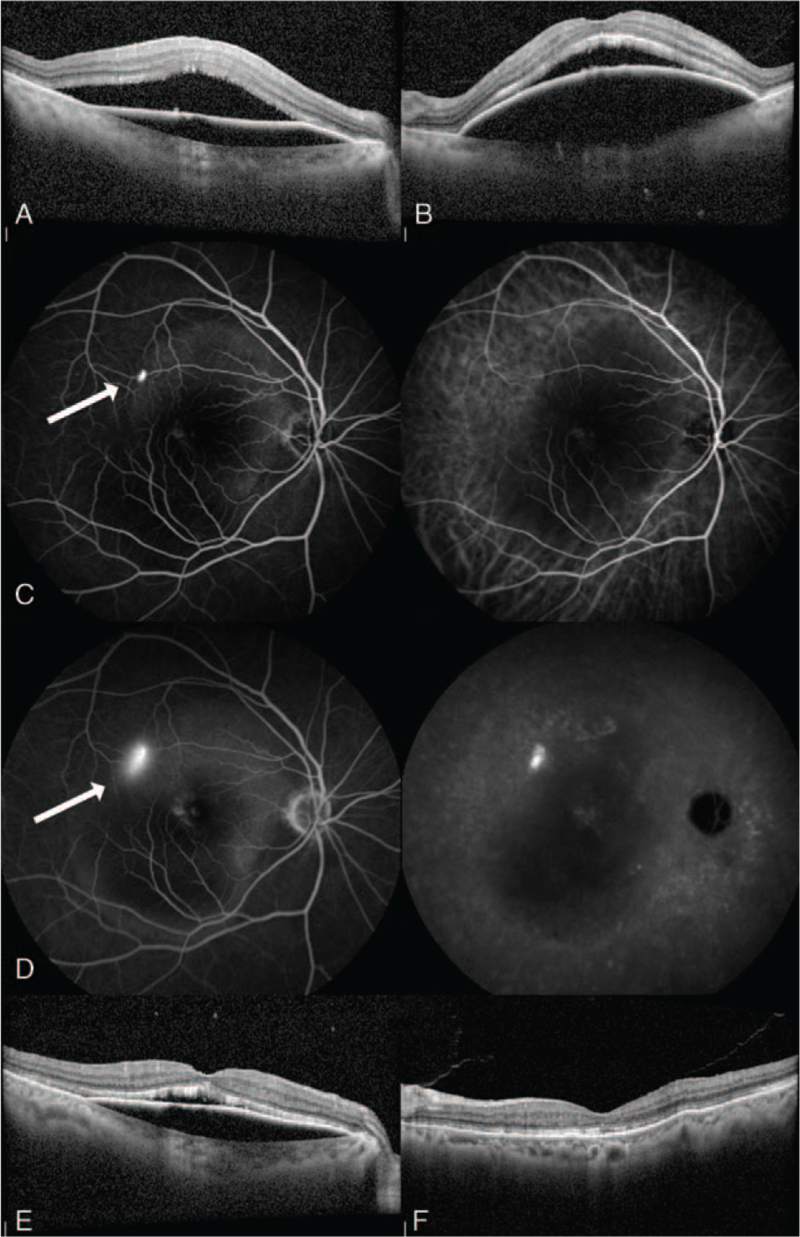
(A, B): Optical coherence tomography (OCT) images from initial examination of right and left eye, respectively. The large size of serous type pigment epithelial detachment (PED) and subretinal fluid (SRF) accumulation were visible on OCT; (C, D): Early and late phase of fluorescence angiography (left) and indocyanine green angiography (right) of the right eye, respectively. White arrows indicate a hyper fluorescence site, and this shows ‘smoke-stack’ sign. The lesion (PED and SRF) size was about 4-disc diameter and was seen as low fluorescence; (E, F): OCT images after 1 month of discontinuation of steroids of right and left eye, respectively. SRF decreased in both eyes. However, PED only improved in the left eye.

Ophthalmologic examinations including fundus photography and optical coherence tomography (OCT) showed asteroid hyalosis, subretinal fluid (SRF), and PED in both eyes (Fig. 1A–B). SRF and PED were located in the macula, with lesions measuring approximately 4-disc diameter. Fluorescence fundus examination including indocyanine green angiography was performed, and showed “smoke-stack” sign (Fig. 1C–D). Characteristic findings of other ophthalmic diseases such as diabetic or hypertensive retinopathy, and choroidal neovascularization membrane (CNVM) were not seen in the examination. The axial length was within the normal range.

We diagnosed steroid-induced acute CSC in both eyes, and hypothesized that consumptions of steroids triggered these large sizes of SRF and PED. Therefore, steroids were discontinued as a treatment for CSC, and a follow-up ophthalmic examination was performed.

**Figure 2:**
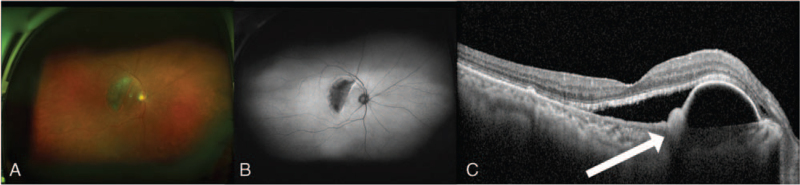
Four months after steroid discontinuation, wide-fundus photography (A), autofluorescence (B), and optical coherence tomography in the right eye were conducted (C). On examination, a crescent shaped retinal pigment epithelial (RPE) tear, about 4-disc diameter in size, was observed. Due to RPE tears, areas without an RPE layer appear to be depigmented (A), and low fluorescence area with hyper fluorescence at the border can be seen (B); (C): The white arrow shows the rolled RPE monolayer with serous PED.

**Figure 3:**
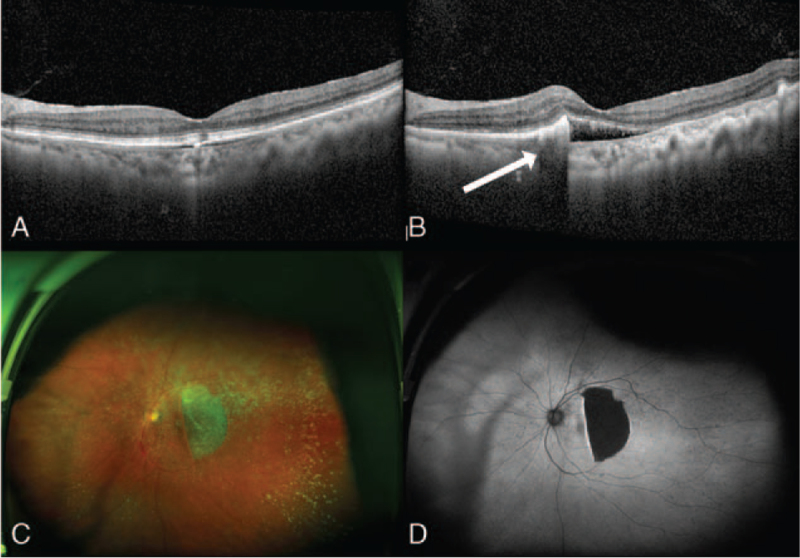
(A): Seven months after steroid discontinuation, optical coherence tomography (OCT) was conducted in the left eye. Shallow and large pigment epithelial detachment (PED) was shown in the image; (B, C, and D): Nine months after steroid discontinuation, OCT, wide-fundus photography, and autofluorescence assessment were conducted. A retinal pigment epithelial (RPE) tear was visible in the previous PED site. The shape and size of the RPE tear was crescent-shaped and about 4-disc diameter, respectively.

After discontinuing the steroids for a month, the SRF decreased, but the PED was improved only in the left eye (Fig. 1E–F). Three months later, the patient visited the ophthalmologist with discomfort in the right eye. Visual acuity in the right eye was 0.3. Slit-lamp examination revealed no abnormal findings in the anterior segment. Fundus examination showed an RPE tear in the previous PED site (Fig. 2A–C). OCT angiography was performed to verify the occurrence of CNVM, but no new vessel was found. As the RPE tear occurred spontaneously even without particular treatment such as focal laser therapy, photodynamic therapy with verteporfin, and anti-VEGF injection, we performed follow-up more frequently than before after notifying the patient that an RPE tear could occur in the opposite eye (Fig. 3A). Despite this, after 5 months, the patient had a spontaneous RPE tear in the left eye as well, with the same size and shape as that of the tear in the right eye (Fig. 3B–D). Visual acuity in the left eye was 0.15, and again CNVM was not found on OCT angiography.

The patient was followed up for an additional 9 months, but there was no change in the RPE tear. Her visual acuity was 0.3 and 0.15 in the right and left eye, respectively. The patient did not experience any specific systemic side effects due to discontinuation of oral steroids.

## 4 Discussion

In this case, the patient acutely developed large PED due to steroid-induced CSC in both eyes. Although the SRF was decreased because of discontinuation of steroids, the large PED spontaneously developed to RPE tears in both eyes sequentially, even though there were no specific CSC treatments such as focal laser, anti-VEGF injection, and photodynamic therapy.

Balaratnasingam et al^[3]^ reported an RPE tear rate of 95% in chronic bullous CSC. However, they did not identify any RPE tears in patients without bullous RD. They also suggested that RPE tears formed in chronic CSC cases were concentric tears with an island of scrolled RPE at the torn center. However, in this case, the patient had an acute CSC without bullous RD. The RPE tears’ shape was large, approximately 4-disc diameter in size, and crescent-shaped rather than concentric, which is different from that previously reported.^[4,7,8]^ Lee et al reported large bilateral RPE tears in a steroid-induced case of CSC, also.^[6]^ However, unlike this case, there was no image data before the RPE tear occurrence, and the shape of the tears was also different.

In this case, the patient had a serous-type large PED without bullous RD due to steroid-induced acute CSC. Steroids are known to increase CSC development by inhibiting the healing of RPE injury and increasing the permeability of the choriocapillaris. Therefore, this injury allows the movement of plasma proteins and water into the subretinal space.^[9]^ In chronic CSC, chronic fibrin accumulation affects to rise of hydrostatic pressure in PED, and this induces concentric-shaped RPE tear (island of scrolled RPE is seen in the center of the RPE site).^[3]^ However, in this case, the shape of the RPE tears was not concentric but crescent-shaped, and the size was also large. In this case, the RPE injury's location, suspected to the RPE tear initiation site, was visible as a “leakage” on fluorescein angiography (Fig. 1C–D, white arrow). Pathophysiology of RPE tear is presumed that advanced age and large size together would have affected the elasticity of the PED, and this mechanical stress above limits induces a micro-rip on the leakage site, and finally, crescent-shape RPE tear occurred. Because there was no chronic fibrin accumulation in PED, the appearance of the RPE tear was not shown like concentric shape.

Crescent-shaped RPE tears are common in AMD.^[1,2]^ RPE tears also can occur after treatment of CNVM by laser photocoagulation, photodynamic therapy with verteporfin, and anti-VEGF injections in patients with AMD.^[2,10,11]^ These treatments are also used for CSC.^[12–14]^ In this case, CNVM was not found on OCT angiography and the patient spontaneously developed RPE tears in both eyes, despite no CSC treatment. Therefore, clinicians should be aware that RPE tears may occur spontaneously in elderly patients with acute CSC and large PED.

Acute CSC, occurring in old age with bilateral eyes, are rare. Therefore, the absence of other similar cases is a limitation of this paper. In the future, more patients will need to be observed.

## 5 Conclusion

In conclusion, we found a spontaneous bilateral large crescent-shaped RPE tear in a case of acute CSC without bullous RD. It is estimated that a large PED and old age may affect RPE tear. Therefore, for an elderly patient with CSC with a large PED, an RPE tear may occur spontaneously, and care must be taken while deciding the treatment.

## Author contributions

**Conceptualization:** Woo Hyuk Lee, Yong Seop Han.

**Formal analysis:** Woo Hyuk Lee.

**Investigation:** Bum Jun Kim.

**Supervision:** Yong Seop Han.

**Writing – original draft:** Woo Hyuk Lee, Yong Seop Han.

**Writing – review & editing:** Woo Hyuk Lee, Yong Seop Han.
